# Coût et facteurs associés à la prescription des médicaments non antirétroviraux chez les patients infectés par le VIH sous thérapie antirétrovirale dans un hÔpital de référence au Maroc

**DOI:** 10.48327/mtsi.2022.199

**Published:** 2022-01-21

**Authors:** Hicham TITOU, Mohammed BOUI, Naoufal HJIRA

**Affiliations:** Hôpital militaire d’instruction Mohammed V, Université Mohammed V, 10100 Rabat, Maroc

**Keywords:** VIH, Thérapie antirétrovirale, Médicaments non antirétroviraux, Ordonnance, Hôpital, Maroc, Maghreb, Afrique du Nord, HIV, Antiretroviral therapy, Non-antiretroviral drugs, Prescription, Hospital, Morocco, Maghreb, Northern Africa

## Abstract

**Objectif:**

Déterminer les coûts des médicaments non antirétroviraux et identifier les facteurs associés à leur prescription chez les patients VIH-1 sous thérapie antiretrovirale au Maroc.

**Méthodes:**

Étude rétrospective d’une cohorte de 264 malades vivant avec le VIH-1 mis sous thérapie antirétrovirale dans le service de dermatologie-vénérologie à l’hôpital militaire d’instruction Mohammed V de Rabat au cours de la période du 1er janvier 2014 au 31 décembre 2018. Les coûts retenus étaient ceux de la pharmacie de l’hôpital pour les médicaments essentiels, sinon le prix de vente en officine. Le modèle de régression logistique a été utilisé pour analyser les facteurs associés à la prescription.

**Résultats:**

Sur les 264 patients inclus, la prédominance masculine était de 75 %. L’âge médian des patients était de 49 ans [41-57]. À l’inclusion, 21,2 % des patients étaient déjà au stade sida. Après une durée moyenne de suivi de traitement antirétroviral estimée à 11,1 ± 6,8 mois, 71,6 % des patients avaient bénéficié d’au moins une prescription de médicament non antirétroviral. Sur toute la durée de suivi, la dépense moyenne par patient était de 24,2 € par an, dont 22,1 € à la charge du patient. Après le cotrimoxazole (30,7 % des patients), les médicaments les plus fréquemment prescrits étaient le fer (29,2 % des patients), les antibiotiques (20,8 % des patients), les hypolipémiants (20,1 % des patients) et les antimycosiques généraux (16,3 % des patients). L’âge (RR: 1,01; IC 95 %: 1,00–1,07), le stade sida (RR: 2,15; IC 95 %: 1,61–4,19), l’anémie (RR: 2,02; IC 95 %: 2,10–5,41) et le nombre de comorbidités (RR: 2,45; IC 95 %: 2,10–5,41) étaient significativement associés à la prescription de médicaments non antirétroviraux.

**Conclusion:**

Notre travail souligne la grande fréquence de la prescription des médicaments non antirétroviraux chez les patients vivant avec le VIH au Maroc; particulièrement à ceux qui sont plus âgés, anémiques à l’inclusion et ceux qui sont déjà au stade sida.

## Abstract (en)

**Objective:**

To determine the costs of non-antiretroviral drugs and to identify the factors associated with their prescription in HIV-1 patients on antiretroviral therapy in Morocco.

**Methods:**

Retrospective study of a cohort of 264 patients living with HIV-1 who were given antiretroviral therapy in the Venerology Dermatology Department at the Mohamed V Military Training Hospital of Rabat during the period from January 1st, 2014, December 31st, 2018. The costs retained were those of the hospital pharmacy for essential drugs, otherwise they were the costs in the private pharmacies. The logistic regression model was used to analyze the factors associated with prescription.

**Results:**

Of the 264 patients included, the male predominance was 75%. The median age of patients was 49 [41-57]. At the onset, 21.2% of patients were already in the AIDS stage. After a mean duration of 11.1 ± 6.8 months of antiretroviral therapy, 71.6% of patients received at least one prescription for a non-antiretroviral drug. Over the entire follow-up period, the mean cost per patient was 24.2 €, and the mean cost supported per patient was 22.1 €. After cotrimoxazole (30.7% of patients), the most frequently prescribed drugs were iron (29.2% of patients), antibiotics (20.8% of patients), hypolipemics (20.1% of patients) and general antimycosics (16.3% of patients). Age (RR: 1.01; 95% CI: 1.00-1.07), AIDS stage (RR: 2.15; 95% CI: 1.61-4.19), anemia (RR: 2.02; 95% CI: 2.10-5.41) and number of comorbidities (RR: 2.45; 95% CI: 2.10-5.41) were significantly associated with the prescription of non-antiretroviral drugs.

**Conclusion:**

Our work highlights the high frequency of prescription of non-antiretroviral drugs in patients living with HIV in Morocco; especially those who are older, anemic at the onset and those who are already at the AIDS stage.

## Introduction

Depuis 2003, la thérapie antirétrovirale (TAR) est de plus en plus généralisée dans les pays en développement (PED). Par conséquent, l’espérance de vie des pvVIH (personnes vivant avec le VIH) a considérablement augmenté, au point que la maladie peut désormais être considérée comme chronique [6]. C’est une victoire à l’échelle mondiale, mais aussi un défi, car il s’accompagnera d’une forte augmentation de la charge des comorbidités et de l’utilisation de médicaments non antirétroviraux (ARV) à mesure que les patients vieillissent [24]. Au Maroc, la prévalence du VIH demeure faible et stable autour de 0,08 %, ce qui caractérise une épidémie de faible intensité [18]. Fin 2019, environ 21 500 personnes vivent avec le VIH (pvVIH) [18]. Parmi elles, on observe une utilisation de la TAR de 76 % et un succès virologique de 70 % [18].

La gratuité et les programmes de lutte multisectoriels et décentralisés ont amélioré l’accès aux services de traitement et de soins dans les PED. Cependant, dans la plupart de ces pays, la prise en charge des pvVIH se limite aux médicaments ARV, les traitements de certaines affections opportunistes ainsi que certains examens biologiques.

Même sous TAR, la prise en charge clinique des pvVIH reste compliquée par la survenue des affections opportunistes, des évènements indésirables liés aux divers médicaments et des complications nutritionnelles et métaboliques. Les pvVIH présentent une prévalence plus élevée de comorbidités apparaissant plus tôt que dans la population générale [24]. Par conséquent, ils prennent généralement plus de médicaments non ARV [24] qui sont déterminants dans le succès de la prise en charge de cette population. Dans les PED, l’accès aux médicaments non ARV devient ainsi plus problématique que l’accès aux ARV pour certains pvVIH.

À notre connaissance, aucune étude évaluant le coût des médicaments non ARV n’a été réalisée au Maghreb. Cette région est géographiquement en continuité avec l’Afrique subsaharienne, qui supporte la part importante du poids de cette pandémie.

L’objectif de cette étude était d’évaluer les coûts des médicaments non ARV et d’identifier les facteurs associés à leur prescription chez les pvVIH au Maroc.

## Matériels et Méthodes

Cette étude analytique a été menée sur une cohorte rétrospective de patients infectés par le VIH de type 1 et suivis dans le service de dermatologie-vénérologie à l’hôpital militaire d’instruction Mohammed V de Rabat. Il s’agit d’un centre de référence régional dans la prise en charge des militaires et de leur famille.

Les critères d’inclusion étaient un âge ≥ 18 ans, une infection VIH-1 confirmée par deux tests ELISA et un test Western Blot, et une TAR débutée entre le 1er janvier 2014 et le 31 décembre 2018. Les combinaisons thérapeutiques reçues étaient AZT (azidothymidine) (ou TDF [ténofovir]) + 3TC + EFV (éfavirenz) (ou IDV [indinavir]). Après initiation de la TAR, le patient devait se présenter en consultation à M1, M2, M3, puis chaque trimestre. Un bilan biologique (hémogramme, transaminases, créatininémie, taux de CD4 et charge virale) était également réalisé tous les semestres.

Nous avons réalisé une analyse anonymisée des données à partir des dossiers médicaux des malades et des registres de la pharmacie: date de début de la TAR, caractéristiques sociodémographiques (âge, sexe, niveau d’instruction), habitudes toxiques (tabagisme, alcoolisme), données cliniques (protocole thérapeutique, stade clinique CDC), données anthropométriques (poids, taille), données biologiques (taux de CD4, taux d’hémoglobine).

Pour les médicaments essentiels, le prix de vente de la pharmacie de l’hôpital militaire d’instruction Mohammed V de Rabat a été retenu. Pour les autres médicaments, le prix de vente en officine a été considéré.

La dépense moyenne par patient en médicaments non ARV correspond à la somme des coûts de tous les médicaments non ARV prescrits au patient durant toute la durée de l’étude. La dépense moyenne par patient en médicaments non ARV payés par le patient correspond à la somme des coûts de tous les médicaments non ARV pris en charge par celui-ci durant toute la durée de l’étude.

## Analyse statistique

L’analyse statistique a été réalisée à l’aide du logiciel Statistical Package for Social Sciences (SPSS) version 22.0. Le modèle de régression logistique a été utilisé pour analyser les facteurs associés à la prescription des médicaments non ARV. Un modèle multivarié initial a été effectué à l’aide des variables avec p < 0,20 en analyse bivariée afin d’identifier les facteurs associés à la prescription des médicaments non ARV. Pour obtenir le modèle final, les interactions dépassant le seuil de 5 % ont été retirées. À chaque étape de l’analyse, le calcul des rapports de cotes (RC) a permis d’estimer les facteurs de confusion. Un intervalle de confiance (95 %) autour du RC contenant la valeur 1 nous a permis de déduire les variables indépendantes. La signification statistique a été définie pour une valeur de p ≤ 0,05.

## Résultats

### Caractéristiques des patients à l’inclusion

Au total, 264 patients ont été inclus dans l’étude, avec une prédominance masculine (75 %). À l’induction de la TAR, l’âge médian des patients était de 49 ans (Intervalle interquartile [IIQ]: 41-57). 21,2 % des patients étaient déjà au stade de sida. Initialement, le taux médian de CD4 était de 297 cellules/ml (IIQ: 112,0-500,7). 37,5 % des patients avaient un taux de CD4 inférieur à 200 cellules/ml. Environ un patient sur 10 (8,3 %) avait un indice de masse corporelle supérieur à 30 kg/m2. Une anémie au-dessous de 10 g/dl a été observée chez 42,8 % des patients. Concernant le protocole thérapeutique à l’inclusion, 16,7 % avaient reçu un protocole contenant de l’indinavir (Tableau I).

**Tableau I:** Caractéristiques cliniques et paracliniques à l’inclusion des 264 patients sous TAR Clinical and paraclinical characteristics of the 264 patients on antiretroviral therapy at onset of study

Caractéristiques	Nombre	%
Âge (ans) médian (IIQ) ≥ 50	49 (41–57) 121	45,8
Sexe masculin féminin	198 66	75 25
Niveau d’instruction aucun primaire secondaire universitaire	137 71 52 4	51,9 26,9 19,6 1,5
Tabagisme jamais ex-tabagique tabagique actuel	131 19 114	49,6 7,2 43,2
Alcoolisme jamais ex-alcoolique alcoolique actuel	169 33 62	64 12,5 23,5
IMC (kg/m2) < 18 18 ≤ IMC< 25 25 ≤ IMC <30 ≥ 30	36 144 62 22	13,6 54,5 23,5 8,3
Stade de CDC à l’induction A B C	178 30 56	67,4 11,4 21,2
Taux d’HG à l’induction ≤ 10 > 10	113 151	42,8 57,2
Taux de CD4 avant l’induction ≥ 200 cellules/ml < 200 cellules/ml	165 99	62,5 37,5
Régime thérapeutique EFV IDV	220 44	83,4 16,7
Présence de comorbidités 0 1 ≥ 2	86 87 91	32,6 33 34,4

IIQ, Intervalle interquartile; IMC, indice de masse corporelle; CDC, Centers for Disease Control and Prevention; HG, hémoglobine; EFV, éfavirenz; IDV, indinavir.

### Coût des médicaments non ARV

La durée moyenne de suivi des patients sous TAR était de 11,1 ± 6,8 mois. Durant cette période, 189 patients (71,6 %) ont bénéficié d’au moins une prescription de médicament non ARV. Le coût total moyen des médicaments non ARV par patient était de 1 280,5 ± 1 479,7 MAD (dirham marocain) (121,2 ± 140,0 €). Les patients bénéficient de la gratuité de certains médicaments non ARV pendant l’hospitalisation, ainsi que pour certaines maladies comme la tuberculose. Ainsi, en prenant en considération cette gratuité partielle, durant la période de suivi, le coût total des médicaments non ARV supporté par patient était de 1 172,3 ± 1 393,4 MAD (110,9 ± 131,8 €). La fréquence de prescription des médicaments non ARV et les dépenses moyennes sont résumées dans le Tableau II.

Après le cotrimoxazole, les médicaments les plus fréquemment prescrits étaient le fer (77 patients), les antibiotiques (55 patients), les hypolipémiants (53 patients) et les antimycosiques généraux (43 patients). Les médicaments non ARV qui revenaient plus cher par patient traité étaient les antimycosiques par voie générale (154,7 €), les antidiabétiques (90,4 €), les antidépresseurs (66,2 €) et les hypolipémiants (62,5 €) (Tableau II).

**Tableau II:** Coût des médicaments non ARV prescrits par classe thérapeutique Cost of prescribed non-antiretroviral drugs by therapeutic class

Médicaments prescrits non ARV	N (%)	Coût moyen (écart-type), (MAD)	Coût moyen supporté par le patient (écart-type), (MAD)
Cotrimoxazole	81 (30,7)	180 (210)	120 (160)
Fer	77 (29,2)	480 (650)	480 (650)
Antibiotiques	55 (20,8)	1040 (1100)	450 (538)
Hypolipémiants	53 (20,1)	1344 (1587)	1344 (1587)
Antimycosiques généraux	43 (16,3)	2200 (2603)	2200 (2603)
Antidiabétiques	42 (15,9)	2016 (2479)	1506 (1981)
Antimycosiques locaux	35 (13,3)	240 (280)	240 (280)
Antidépresseurs	23 (8,7)	2225 (2678)	2225 (2678)
Vitamines	20 (7,6)	967 (1090)	967 (1090)
Antiulcéreux	16 (6,1)	1090 (1207)	1090 (1207)
Antihypertenseurs	7 (2,7)	3500 (3743)	4500 (5743)

MAD, Dirham marocain

### Facteurs associés à la prescription

Parmi les caractéristiques à l’inclusion, l’âge (RR = 1,01; IC 95 %: 1,00–1,07), le stade sida (RR = 2,15; IC 95 %: 1,61–4,19), l’anémie (RR: 2,02; IC 95 %: 2,10–5,41) et le nombre de comorbidités (RR = 2,45; IC 95 %: 2,10–5,41) étaient associés de façon significative à la prescription de médicaments non ARV (Tableau III).

**Tableau III:** Analyse univariée et multivariée en régression logistique des facteurs associés à la prescription de médicaments non ARV Univariate and multivariate analysis using logistic regression of factors associated with the prescription of non-antiretroviral drugs

Caractéristiques	Analyse univariée		Analyse multivariée	
	p	RC (IC 95 %)	p	RC (IC 95 %)
Âge (ans)	0,001	1,04 (1,01–1,07)	0,01	1,03 (1,00–1,07)
Sexe masculin féminin	1 0,58	0,83 (0,44–1,57)	–	–
Tabagisme, n (%) jamais ex-tabagique tabagique actuel	1 0,22 0,73	0,54 (0,20–1,45) 1,10 (0,62–1,93)	–	–
Alcoolisme, n (%) jamais ex-alcoolique alcoolique actuel	1 0,93 0,42	0,96 (0,42–2,17) 1,31 (0,67–2,56)	–	–
Obésité	0,42	0,61 (0,18–2,01)	–	–
Stade de CDC à l’induction, n (%) A B C	1 0,05 0,005	1,98 (0,80–4,87) 2,62 (1,61–4,12)	1 0,09 0,008	2,16 (0,80–4,87) 2,15 (1,61–4,19)
Taux d’HG à l’induction	< 0,001	3,06 (2,48–4,55)	< 0,001	2,02 (2,10–4,41)
Régime thérapeutique, n (%) EFV IDV	1 0,08	2,03 (0,90–2,61)	0,27	1,64(0,67–3,41)
Présence de comorbidités 0 1 ≥ 2	0,007 < 0,001	2,3 (1,26–3,42) 3,5 (3,20–4,56)	0,01 < 0,001	2,45 (1,34–3,78) 3,89 (3,43–4,87)

RC, rapport de cotes; CDC, Centers for Disease Control and Prevention; HG, hémoglobine; EFV, éfavirenz; IDV, indinavir.

## Discussion

Notre travail rapporte un suivi de 11,1 mois d’une cohorte de 264 patients infectés par le VIH-1, sous TAR entre 2014 et 2018. Dans un souci d’exhaustivité, nous avons collecté les données à partir de deux sources (dossiers médicaux et données administratives). Nos résultats ont objectivé une haute fréquence (71,6 %) de la prescription de médicaments non ARV. Leur coût moyen était de 1 280,5 MAD dont 1 172,3 MAD à la charge du patient. Les facteurs associés à cette prescription étaient l’âge, le stade sida, l’anémie et le nombre de comorbidités.

Le caractère rétrospectif de notre étude comporte des limites liées à l’accessibilité et à la complétude des dossiers médicaux. L’absence d’une comparaison avec une population témoin constitue une autre limite de cette étude. Cependant, notre analyse est la première à évaluer les besoins véritables des pvVIH et les facteurs associés à la prescription des médicaments non ARV en Afrique du Nord.

Dans la présente étude, le coût moyen/patient/an était de 24,2 €. Par rapport à notre résultat, des études antérieures ont montré un coût moyen plus bas au Sénégal [7] et au Kenya [12] et d’autres études ont trouvé un coût moyen plus important en Afrique du Sud [13] et au Rwanda [19]. La discordance entre ces résultats peut être attribuée en partie aux fluctuations du coût des médicaments et aux habitudes des praticiens concernant la prescription de médicaments génériques [10, 28]. Le coût moyen des traitements non ARV augmente avec la durée de l’infection et représente le coût le plus important après la mortalité chez les pvVIH [21, 23, 30]. En effet, l’allongement de l’espérance de vie grâce à la généralisation de la TAR a contribué à l’augmentation des dépenses en coûts directs [4, 23]. Cependant, des recherches antérieures ont suggéré que l’adoption universelle de la TAR pourrait entraîner une baisse des dépenses indirectes en améliorant la productivité économique chez les pvVIH [11]. Au Maroc, le salaire moyen, tous secteurs confondus, était de 6 333 MAD (563 €) en mai 2018. Considéré comme insuffisant, le salaire minimum agricole garanti (SMIG) était de 2 566 MAD/mois (242 €) en 2018 [15]. En 2014, l’Enquête nationale sur la consommation et les dépenses des ménages a révélé que les Marocains dépensaient moins de 15 900 MAD (1513,83 €)/personne/an. Le poids de l’hygiène et des soins médicaux dans le budget global est de 8,7 % (131 70 €) [22]. Notre étude a montré qu’un Marocain séropositif dépensait annuellement 24,2 € en médicaments non ARV, dépassant de 18 % le budget annuel consacré à l’hygiène et aux soins médicaux dans la population générale (116,4€). Bien que s’intéressant à l’estimation du coût des médicaments non ARV, cette étude n’a pas pris en compte d’autres coûts associés directement ou non aux médicaments non ARV comme le transport, la consultation, l’accompagnement du patient, les heures de travail perdues et les effets indésirables des médicaments ARV. Au Maroc, seulement 20,5 % de la population est couverte par au moins un système de protection sociale permettant un remboursement, souvent partiel, des dépenses de santé. Cela explique que les coûts directs des soins sont le plus souvent à la charge des patients. Eu égard aux capacités financières de la population générale du Maroc et au caractère chronique de l’infection VIH, il semblerait que les dépenses directes annuelles moyennes de santé liées à cette infection placent les ménages en situation de « dépenses catastrophiques » [3].

Après le cotrimoxazole, les médicaments les plus fréquemment prescrits étaient le fer, les antibiotiques, les hypolipémiants et les antimycosiques généraux. Plusieurs études ont rapporté un résultat similaire pour les anti-infectieux (antifongiques, antibiotiques, antiparasitaires) [7, 17]. Concernant la fréquence de la prescription des hypolipémiants, nos résultats concordent avec ceux des études antérieures [22, 30]. La plupart des études réalisées en pays développé ont rapporté une grande fréquence de la prescription des médicaments cardiovasculaires [8, 9, 26].

Les facteurs associés de façon indépendante à la prescription de médicaments non ARV dans notre cohorte étaient l’âge, le stade sida à l’induction, l’anémie et le nombre de comorbidités.

Dans notre étude, l’âge avancé était un facteur associé à la prescription de médicaments non ARV. Des études antérieures ont estimé que d’ici 2030, plus de 70 % des pvVIH seront âgés de plus de 50 ans [25]. À mesure que l’espérance de vie s’est accrue, l’incidence estimée des comorbidités associées à la TAR et à l’âge a considérablement augmenté, entraînant une majoration importante des dépenses totales de l’infection VIH. Les interventions réduisant l’exposition cumulée aux ARV peuvent contribuer à réduire la prévalence de ses toxicités. Les recherches futures devraient être axées sur l’évaluation de l’impact de l’exposition réduite aux ARV sur le coût total durant toute la vie des pvVIH.

Dans la présente étude, le nombre de comorbidités était un facteur associé au risque de la prescription de médicaments non ARV. Certaines études ont estimé que, d’ici 2030, environ un tiers des pvVIH sera diagnostiqué avec au moins 3 comorbidités en plus de l’infection VIH [1]. Par conséquent, 40 % des pvVIH subiront probablement des complications liées à des contre-indications ou des interactions médicamenteuses entre les médicaments non ARV et la TAR [24]. La prévalence de la polypharmacie chez les pvVIH était plus élevée que chez les patients séronégatifs dans les différentes catégories d’âge [14, 20]. Ritchwood et al. ont rapporté que le coût total de la prise en charge de comorbidités chez les pvVIH était supérieur à celui de comorbidités chez les patients séronégatifs [21].

Dans notre étude, le stade sida à l’induction était un facteur associé à la prescription de médicaments non ARV. Dans la presque totalité des études antérieures, le coût de la prise en charge des pvVIH était d’autant plus élevé que le stade clinique était avancé, ce qui est concordant avec nos résultats [5, 7, 13]. En effet, dans notre centre, le stade clinique CDC (Centers for Disease Control and Prevention) avancé (stade C) était un facteur associé au risque de mortalité [27]. Contrairement à d’autres études menées en Afrique subsaharienne, le taux de CD4 n’a pas été associé à une augmentation du coût de la prise en charge des pvVIH [2, 13, 15]. En effet, la courte durée de suivi et la faible concentration des taux de CD4 en dessous de 200 cellules/ml dans notre cohorte pourraient expliquer notre résultat.

Nous n’avons trouvé aucune étude dans la littérature qui se soit intéressée à la relation entre l’anémie et le coût des médicaments non ARV. Bien qu’aucune relation de cause à effet n’ait été prouvée, des études antérieures ont rapporté une corrélation entre l’anémie à l’induction et la diminution de la survie et l’augmentation de la progression de la maladie chez les pvVIH [16].

Au Maroc, la prise en charge de l’infection à VIH a bénéficié d’une mobilisation de ressources domestiques et extérieures, en particulier le Fonds mondial (35 % du budget). Les médicaments ARV, les explorations immuno-virologiques et les traitements de certaines affections opportunistes sont dispensés gratuitement, mais restent centralisés. En cas d’affection intercurrente ou d’effets indésirables des ARV, le transport, la consultation, les examens complémentaires, les médicaments non ARV et les hospitalisations sont à la charge du patient. Le montant élevé de ces frais peut avoir un impact négatif sur l’accès aux soins, l’adhésion aux traitements, et donc sur l’efficacité de la prise en charge.

La présente étude fournit des estimations du coût de ces médicaments chez les pvVIH en utilisant les données d’un échantillon représentatif au niveau national. Nos résultats pourraient susciter l’élaboration de politiques budgétaires envisageant la gratuité totale des dépenses directes et en particulier l’accès aux médicaments non ARV chez les pvVIHpvVIH. La plus grande partie de ces dépenses résulte de la prescription de médicaments non ARV [10, 21, 23]. Le paiement direct des soins constitue un frein à la bonne prise en charge des personnes vivant avec le VIH [29]. Cependant, ces coûts apparaissent suffisamment faibles pour qu’ils puissent être couverts par les programmes de prise en charge du VIH ou intégrés aux dispositifs de couverture médicale dans les PED.

## Conclusion

À mesure que les pvVIH vieillissent au Maroc, les dépenses directes augmentent, en particulier le coût des médicaments non ARV. Cette charge financière peut être un obstacle à une prise en charge correcte de ces patients. Or, ces coûts peuvent être largement supportés par les systèmes de protection sociale. La gratuité totale des médicaments non ARV pour les pvVIH devrait être au centre des politiques et des programmes nationaux.

## Liens d’intérêts

Les auteurs ne déclarent aucun lien d’intérêt.

## Contribution des auteurs

Conception de l’étude, recueil des données, rédaction du manuscrit: Hicham TITOU. Définition de la méthodologie, analyse des données: Naoufal HJIRA. Relecture, correction et validation du manuscrit: Mohammed BOUI

## References

[1] Althoff KN, Smit M, Reiss P, Justice AC (2016). HIV and ageing: improving quantity and quality of life. Curr Opin HIV AIDS.

[2] Barnett PG, Chow A, Joyce VR, Bayoumi AM, Griffin SC, Nosyk B, Holodniy M, Brown ST, Sculpher M, Anis AH, Owens DK (2011). Determinants of the cost of health services used by veterans with HIV. Med Care.

[3] Boufkhed S, Taverne B (2014). Évaluation du coût direct de la prise en charge médicale du VIH entre la troisième et la dixième année de traitement ARV à Dakar. Bull Soc Pathol Exot.

[4] Bozzette SA, Joyce G, McCaffrey DF, Leibowitz AA, Morton SC, Berry SH, Rastegar A, Timberlake D, Shapiro MF, Goldman DP (2001). HIV Cost and Services Utilization Study Consortium. Expenditures for the care of HIV-infected patients in the era of highly active antiretroviral therapy. N Engl J Med.

[5] Decock RC, Depoorter AM, De Graeve D, Colebunders R (2001). Direct costs of health care for HIV/AIDS patients in Belgium. AIDS Care.

[6] Deeks SG, Lewin SR, Havlir DV (2013). The end of AIDS: HIV infection as a chronic disease. Lancet.

[7] Diouf A, Youbong TJ, Maynart M, Ndoye M, Diéye FL, Ndiaye NA, Koita-Fall MB, Ndiaye B, Seydi M (2017). Médicaments non antirétroviraux chez les personnes vivant avec le VIH sous traitement antirétroviral au Sénégal: coûts et facteurs associés à la prescription [Non-antiretroviral drugs uses among HIV-infected persons receiving antiretroviral therapy in Senegal: Costs and factors associated with prescription]. Rev Epidemiol Sante Publique.

[8] Edelman EJ, Rentsch CT, Justice AC (2020). Polypharmacy in HIV: recent insights and future directions. Curr Opin HIV AIDS.

[9] El Moussaoui M, Lambert I, Maes N, Sauvage AS, Frippiat F, Meuris C, Uurlings F, Lecomte M, Léonard P, Fombellida K, Vaira D, Vercheval C, Moutschen M, Darcis G (2020). Evolution of Drug Interactions With Antiretroviral Medication in People With HIV. Open Forum Infect Dis.

[10] Gebo KA, Chaisson RE, Folkemer JG, Bartlett JG, Moore RD (1999). Costs of HIV medical care in the era of highly active antiretroviral therapy. AIDS.

[11] Hutchinson AB, Farnham PG, Dean HD, Ekwueme DU, del Rio C, Kamimoto L, Kellerman SE (2006). The economic burden of HIV in the United States in the era of highly active antiretroviral therapy: evidence of continuing racial and ethnic differences. J Acquir Immune Defic Syndr.

[12] Larson BA, Bii M, Henly-Thomas S, McCoy K, Sawe F, Shaffer D, Rosen S (2013). ART treatment costs and retention in care in Kenya: a cohort study in three rural outpatient clinics. J Int AIDS Soc.

[13] Leisegang R, Cleary S, Hislop M, Davidse A, Regensberg L, Little F, Maartens G (2009). Early and late direct costs in a Southern African antiretroviral treatment programme: a retrospective cohort analysis. PLoS Med.

[14] López-Centeno B, Badenes-Olmedo C, Mataix-Sanjuan Á, McAllister K, Bellón JM, Gibbons S, Balsalobre P, Pérez-Latorre L, Benedí J, Marzolini C, Aranguren-Oyarzábal A, Khoo S, Calvo-Alcántara MJ, Berenguer J (2020). Polypharmacy and Drug-Drug Interactions in People Living With Human Immunodeficiency Virus in the Region of Madrid, Spain: A Population-Based Study. Clin Infect Dis.

[15] (2020). Ministère du Travail et de l’Insertion Professionnelle. Le marché du travail en 2019. Maroc.

[16] Mocroft A, Kirk O, Barton SE, Dietrich M, Proenca R, Colebunders R, Pradier C, d’Arminio Monforte A, Ledergerber B, Lundgren JD (1999). Anaemia is an independent predictive marker for clinical prognosis in HIV-infected patients from across Europe. EuroSIDA study group. AIDS.

[17] Nombela N, Kouadio B, Toure S, Seyler C, Flori YA, Anglaret X (2006). Nonantiretroviral drug consumption by CD4 cell count in HIV-infected adults: a 5-year cohort study in Côte d’Ivoire. J Acquir Immune Defic Syndr.

[18] Onusida (2020). Le sida en chiffres. https://www.unaids.org/sites/default/files/media_asset/UNAIDS_FactSheet_fr.pdf.

[19] Quentin W, König HH, Schmidt JO, Kalk A (2008). Recurrent costs of HIV/AIDS-related health services in Rwanda: implications for financing. Trop Med Int Health.

[20] Rasmussen LD, Kronborg G, Larsen CS, Pedersen C, Gerstoft J, Obel N, Pottegård A (2017). Use of non-antiretroviral drugs among individuals with and without HIV-infection: a Danish nationwide study. Infect Dis (Lond).

[21] Ritchwood TD, Bishu KG, Egede LE (2017). Trends in healthcare expenditure among people living with HIV/AIDS in the United States: evidence from 10 years of nationally representative data. Int J Equity Health.

[22] Royaume du Maroc, Haut-Commissariat au Plan. Enquête nationale sur la consommation et les dépenses des ménages 2013/2014. Rapport de synthèse.

[23] Schackman BR, Fleishman JA, Su AE, Berkowitz BK, Moore RD, Walensky RP, Becker JE, Voss C, Paltiel AD, Weinstein MC, Freedberg KA, Gebo KA, Losina E (2015). The lifetime medical cost savings from preventing HIV in the United States. Med Care.

[24] Smit M, Brinkman K, Geerlings S, Smit C, Thyagarajan K, Sighem Av, de Wolf F, Hallett TB (2015). ATHENA observational cohort. Future challenges for clinical care of an ageing population infected with HIV: a modelling study. Lancet Infect Dis.

[25] Smit M, Cassidy R, Cozzi-Lepri A, Quiros-Roldan E, Girardi E, Mammone A, Antinori A, Saracino A, Bai F, Rusconi S, Magnani G, Castelli F, Hsue P, d’Arminio Monforte A, Hallett TB (2017). Projections of non-communicable disease and health care costs among HIV-positive persons in Italy and the U.S.A.: A modelling study. PLoS One.

[26] Sung M, Gordon K, Edelman EJ, Akgün KM, Oursler KK, Justice AC (2021). Polypharmacy and frailty among persons with HIV. AIDS Care.

[27] Titou H, Baba N, Kasouati J, Oumakir S, Frikh R, Boui M, Hjira N (2018). Survie des patients vivant avec le VIH-1 sous thérapie antirétrovirale au Maroc. Rev Epidemiol Sante Publique.

[28] Walensky RP, Sax PE, Nakamura YM, Weinstein MC, Pei PP, Freedberg KA, Paltiel AD, Schackman BR (2013). Economic savings versus health losses: the cost-effectiveness of generic antiretroviral therapy in the United States. Ann Intern Med.

[29] Whiteside A, Lee S (2005). The «free by 5» campaign for universal, free antiretroviral therapy. PLoS Med.

[30] Yang CJ, Wang HY, Chou TC, Chang CJ (2019). Prevalence and related drug cost of comorbidities in HIV-infected patients receiving highly active antiretroviral therapy in Taiwan: A cross-sectional study. J Microbiol Immunol Infect.

